# Pharmacological Interventions in Autism Spectrum Disorder: A Comprehensive Review of Mechanisms and Efficacy

**DOI:** 10.3390/biomedicines13123025

**Published:** 2025-12-10

**Authors:** Eva Sclabassi, Sophie Peret, Chunqi Qian, Yuen Gao

**Affiliations:** Department of Radiology, Michigan State University, East Lansing, MI 48824, USA; sclabas6@msu.edu (E.S.); peretsop@msu.edu (S.P.); qianchu1@msu.edu (C.Q.)

**Keywords:** autism spectrum disorder, pharmacology, antipsychotics, selective serotonin reuptake inhibitors, glutamatergic agents, neuroinflammation, oxidative stress, personalized medicine

## Abstract

**Background and Objectives:** Autism spectrum disorder (ASD) is a heterogeneous neurodevelopmental condition characterized by social communication deficits, restricted interests, and repetitive behaviors. At present, there is no pharmacological intervention that reliably targets the core symptoms of ASD; instead, medications are primarily used to manage associated or concurrent symptoms such as irritability, aggression, anxiety, attention difficulties, and sleep disturbances. This review summarizes the current evidence for pharmacological treatments in ASD, emphasizing how these interventions are used in a symptom-focused, adjunctive manner, and highlighting efficacy, mechanisms, limitations, and emerging therapeutic targets. **Methods:** A comprehensive literature review was conducted across PubMed, Cochrane Library, and Embase to identify clinical trials, systematic reviews, meta-analyses, and preclinical studies on pharmacological interventions for ASD. Seventy-seven references were integrated to reflect the current state of evidence. **Results:** Established pharmacological strategies include atypical antipsychotics for severe irritability and aggression, as well as antidepressants, stimulants and non-stimulant agents, mood stabilizers, and anxiolytics for selected comorbid symptoms, although efficacy is often modest and variable, and side effects can be significant. Adjunctive and investigational approaches targeting glutamatergic and GABAergic neurotransmission, monoaminergic systems, and neuroinflammatory and oxidative stress pathways show preliminary promise but remain experimental. Across all categories, pharmacological treatments are most effective when embedded in individualized, multimodal care plans that integrate behavioral, rehabilitative, and psychological interventions. **Conclusions:** This review maps pharmacologic strategies in ASD onto their underlying neurobiological mechanisms and clarifies how evidence strength differs across drug classes and symptom domains. Ongoing advances in genetics, synaptic and circuit-level neuroscience, and neuroimmune signaling are expected to yield more specific, mechanism-based pharmacological approaches for autistic behaviors, with the potential to improve long-term functioning and quality of life when combined with comprehensive psychosocial care.

## 1. Introduction

ASD is a complex neurodevelopmental condition defined by persistent deficits in social communication and restricted, repetitive patterns of behavior. In addition to behavioral presentation, ASD is associated with alterations in excitatory–inhibitory neurotransmission and neuroimmune interactions, which provide a neurobiological basis for several emerging pharmacologic targets. Recent estimates from the U.S. Centers for Disease Control and Prevention indicate a prevalence of approximately 1 in 36 children [1]. Contemporary diagnostic frameworks emphasize the spectrum and dimensional nature of ASD. Earlier neurological systems distinguished between autistic disorder, Asperger syndrome, and pervasive developmental disorder—not otherwise specified, but DSM-5 and DSM-5-TR now group these presentations under a single spectrum diagnosis, with specifiers for intellectual level, language abilities, and associated medical or psychiatric conditions [2,3,4]. This shift reflects accumulating evidence that autistic traits are continuously distributed, and that social-communication differences, restricted interests, sensory profiles, and adaptive functioning vary in severity rather than forming discrete categories [2,3,4]. Neuroimaging and neuropathological studies support this dimensional perspective, demonstrating widespread alterations in cortical connectivity, brain growth trajectories, and synaptic organization rather than focal lesions [5,6].

Etiopathogenetically, ASD is now understood as arising from complex interactions between genetic vulnerability and environmental influences. Family, twin, and population-based studies show substantial heritability and increased familial recurrence, and molecular work has implicated rare de novo mutations, copy-number variants, and common polygenic risk in ASD liability [2,3,7,8]. Prenatal and perinatal risk factors—including maternal immune activation, metabolic and inflammatory disturbances, obstetric complications, and certain environmental exposures—may further disrupt neurodevelopmental trajectories, particularly in genetically susceptible individuals [2,4,7]. At the mechanistic level, converging data implicate alterations in synaptic plasticity, excitatory–inhibitory balance, neuroimmune signaling, oxidative stress responses, mitochondrial function, and neural circuit organization [5,6,9,10,11,12]. These interacting pathways help explain the marked heterogeneity of ASD phenotypes and provide biological targets for current and emerging pharmacological strategies.

Large-scale surveys have confirmed that psychotropic medication use is common in individuals with ASD, reflecting the high prevalence of comorbid symptoms such as anxiety, aggression, hyperactivity, and seizures [13,14]. Beyond these symptoms, ASD is frequently accompanied by a wide range of co-occurring psychiatric, neurological, and medical conditions that influence prognosis and quality of life. Anxiety disorders, attention-deficit/hyperactivity disorder, mood disorders, obsessive–compulsive symptoms, disruptive irritability, and self-injurious behavior are all more prevalent in autistic populations than in non-autistic peers and often drive psycho-tropic prescribing [2,3,4,15]. Epilepsy and subclinical epileptiform activity occur at elevated rates and have been associated with greater behavioral complexity and functional impairment [16,17,18]. Sleep disturbances, gastrointestinal complaints, feeding and eating difficulties, sensory hypersensitivities, and putative immune–metabolic abnormalities further contribute to clinical heterogeneity and caregiver burden [11,12,17,18,19]. These comorbidities underscore that pharmacotherapy in ASD is usually directed toward specific symptom clusters rather than toward core diagnostic traits alone. Despite expanding pharmacological research, there is currently no medication approved to treat the core social-communication differences or the restricted and repetitive behaviors that define ASD. Available psychotropic agents therefore provide symptomatic rather than disease-modifying benefit and are primarily used to target co-occurring irritability, aggression, anxiety, depression, hyperactivity, sleep disturbance, and seizure disorders [13,14,17,18,20,21,22,23,24,25,26,27,28,29,30,31,32,33,34]. As a result, pharmacotherapy is often empirical and guided by specific target symptoms, co-occurring diagnoses, and tolerability considerations instead of directly modifying the underlying neurodevelopmental processes that give rise to ASD [2,3,35,36].

The International Society for Autism Research (INSAR) guidelines and other consensus statements emphasize the importance of integrating pharmacological treatments with behavioral and educational approaches but also highlight the absence of a single universally effective medication [16]. This variability reflects underlying heterogeneity in phenotype expression, co-occurring conditions such as ADHD and anxiety, and differences in neurochemical and genetic profiles across individuals with ASD. Consistent with these limitations, international guidelines and expert consensus statements emphasize that behavioral and educational interventions are the foundation of ASD care, particularly in children [2,3,35,36,37,38]. Structured behavioral programs, interpersonal therapies, cognitive-behavioral approaches adapted for autism, speech and occupational therapy, and parent-mediated interventions are considered first-line for addressing core social-communication differences and restrictive, repetitive patterns of behavior [2,3,4,37]. Digital and technology-assisted rehabilitation tools are increasingly used to extend access and support generalization of skills. In contrast, psychoactive medications are generally reserved for clearly defined, impairing co-occurring symptoms—such as severe irritability, anxiety, hyperactivity, or sleep disturbance—and are used as adjuncts to, rather than replacements for, behavioral and rehabilitative interventions [33,34,35,36,37,38].

The heterogeneity of ASD presentations contributes to the diversity of pharmacological strategies employed. Epidemiological research suggests that up to two-thirds of individuals with ASD are prescribed at least one psychotropic drug during their lifetime [13,15]. Observational studies demonstrate that antipsychotics, stimulants, antidepressants, and anticonvulsants are the most frequently used classes, though prescribing practices vary by age, severity of symptoms, and access to services [19,35]. Prescribing patterns also vary geographically, with higher off-label SSRI and antipsychotic use reported in the U.S. compared with Europe and Asia, underscoring differences in regulatory approval and clinical practice. Additionally, some medications discussed in this review are used off label in ASD, and their evidence base differs accordingly. The objective of this review is to map pharmacologic targets to underlying neurobiological mechanisms and evaluate the relative strength of evidence across drug classes, while identifying areas where translational development is still needed. To support clinical applicability, each medication discussed below is accompanied by clarification of the symptom domains and ASD subgroups for which the medication is most used, as well as considerations for combined treatment strategies when clinically appropriate.

The primary aim of this review is to map pharmacologic treatments in ASD to their underlying neurobiological mechanisms, while clarifying the specific symptom domains and clinical contexts in which each medication class may be most appropriate. This review is conducted as a narrative synthesis of the current pharmacologic interventions studied in ASD. Literature was identified through PubMed, Scopus, and Web of Science searches using terms autism, ASD, pharmacological treatment, psychotropic medications, neurotransmitters, immune pathways, and emerging therapies. Studies published between 2000 and 2025 were prioritized to capture both long-established and current treatment evidence. Randomized controlled trials, systematic reviews, and meta-analyses were emphasized when available, with observational and mechanistic studies included when higher-level evidence was limited. Mechanistic categories were informed by consistent findings on neurotransmitter dysregulation, neuroinflammation, and oxidative stress biology in ASD [39]. Given the heterogeneity of study designs, populations, and outcome measures, a narrative synthesis approach was selected instead of meta-analysis. This approach allows for clear mapping between neurobiological rationale and clinical decision-making while maintaining emphasis on individualized treatment selection.

**Part I. Medications Currently Used in Clinical Practice** 

Pharmacologic treatment in ASD remains symptom-focused, with no medications currently approved for improving core social communication difficulties. Instead, clinicians use several drug classes to target common co-occurring symptoms such as irritability, aggression, anxiety, hyperactivity, sleep disturbance, and mood instability (Table 1). Collectively, these medications represent the current evidence-supported options for managing behavioral and functional challenges in ASD, each with distinct mechanisms and symptom-domain targets.

## 2. SSRIs and Serotonergic Modulation

Target symptoms: anxiety, obsessive-compulsive behaviors, repetitive actions, mood dysregulation.

### 2.1. Mechanistic Rationale

Serotonergic dysregulation has long been implicated in ASD pathophysiology. Early neuroimaging studies demonstrated alterations in serotonin synthesis capacity in children with autism [39]. Preclinical and translational studies further suggested that modulation of the serotonin transporter (SERT) could normalize aspects of repetitive behavior [40]. These findings provided a rationale for the clinical use of SSRIs. Altered serotonin (5-HT) synthesis is not a simple primary cause or a clear-cut secondary effect, but rather part of complex, reciprocal interactions within a larger system, influenced by genetic and environmental factors [71]. The intricate relationship involves significant “crosstalk” with norepinephrine and dopamine systems, making a strictly linear primary versus secondary distinction difficult [72]. Genetic predispositions, such as polymorphisms in the TPH2, 5-HTT, and 5-HT_2_A/5-HT_1_A receptor genes, affect the system’s development and function [73,74].

### 2.2. Clinical Evidence

Clinical evidence for SSRIs in ASD is mixed. The Cochrane review by Williams et al. synthesized randomized trials and concluded that SSRIs showed limited benefits in children but may have utility for adults, particularly in managing anxiety and obsessive–compulsive symptoms [41]. Subsequent randomized controlled trials, such as the fluoxetine study by Reddihough et al., demonstrated reductions in obsessive–compulsive behaviors in youth, though adverse effects such as irritability and sleep disturbance were more frequent compared with placebo [42].

Observational research highlights that SSRIs remain one of the most prescribed medication classes for individuals with ASD, particularly in the context of comorbid anxiety [37]. Jobski et al. and Coleman et al. reported frequent parental endorsement of SSRIs in large national surveys [13,14]. Across clinical studies, sample sizes typically range from approximately 20 to 150 participants, with benefits more consistently observed for anxiety and obsessive–compulsive behaviors than for core social communication symptoms.

### 2.3. Preclinical Insights

Animal studies have contributed to mechanistic insights. Golub et al. found that juvenile rhesus monkeys treated with fluoxetine demonstrated increased social interaction [43], while He et al. used metabolomic profiling to identify individual predictors of fluoxetine response, suggesting biological heterogeneity in treatment effects [44]. Rhesus monkey and metabolomic studies provide valuable insight into serotonergic contributions to social behavior; however, these models more accurately reflect serotonin-linked social deficits rather than the full spectrum of ASD pathology [75,76]. Moreover, preclinical evidence shows that early developmental SSRI exposure can lead to long-lasting behavioral changes—including altered social interaction and anxiety—underscoring the developmental sensitivity of the serotonin system and the need for caution when translating these findings to ASD [77,78].

Other SSRIs, such as citalopram and sertraline, have been tested, though results remain inconclusive. King et al. reported that citalopram did not outperform placebo in reducing repetitive behaviors in children with ASD [45], while smaller sertraline trials suggested potential benefit for comorbid anxiety symptoms [46]. Escitalopram has been evaluated in limited pediatric and adult samples, with mixed findings and variable tolerability across individuals [79]. Fluvoxamine has demonstrated some benefit for obsessive-compulsive symptoms, particularly in adolescents and adults, although effects are inconsistent in younger children [80]. Across these agents, dose–response patterns are not well established, and clinical response appears heterogeneous [81].

Beyond their role in managing hyperactivity and impulsivity, pharmacologic strategies for sleep disturbance are a frequent component of ASD care. Insomnia and fragmented sleep can exacerbate daytime irritability, inattention, and self-injurious behavior, and they significantly increase caregiver stress [2,3,19]. Melatonin is the best-studied agent for insomnia in ASD, with randomized trials demonstrating improvements in sleep-onset latency and, in some cases, total sleep time, alongside a favorable tolerability profile [18]. In clinical practice, melatonin is often combined with behavioral sleep hygiene interventions [82].

Sedating antidepressants such as trazodone or mirtazapine, and α_2_-adrenergic agonists such as clonidine, are sometimes used off-label for more refractory insomnia or when anxiety and hyperarousal are prominent, though controlled data in autistic populations remain limited [52,53,67]. Given the interaction between sleep, behavior, and learning, pharmacologic management of insomnia should be regularly re-evaluated and closely integrated with behavioral and environmental strategies.

Feeding and eating difficulties—including sensory-based food selectivity, rigid food preferences, reduced appetite, and feeding-related distress—are also common in ASD and can contribute to growth concerns and micronutrient deficiencies [2,3]. Management primarily relies on behavioral feeding interventions and dietetic support, but pharmacologic options are sometimes considered as adjuncts. Cyproheptadine, an antihistamine with antiserotonergic and appetite-stimulating properties, is frequently used off-label to promote weight gain in children with markedly reduced intake, though robust ASD-specific trial data are limited and much of the evidence comes from broader pediatric populations. When feeding difficulties are exacerbated by gastrointestinal symptoms such as reflux, constipation, or motility disturbances, acid-suppressing agents, laxatives, or prokinetic medications may be used as part of a multidisciplinary plan [18]. Optimizing nutritional status can indirectly improve energy, participation in therapies, and overall health, highlighting the interconnectedness of metabolic, gastrointestinal, and behavioral domains in ASD.

### 2.4. Clinical Considerations

Taken together, SSRIs may alleviate specific domains such as anxiety and obsessive–compulsive behaviors, but effects on the core features of ASD remain limited. Clinical use requires careful dose titration and monitoring for adverse events such as gastrointestinal upset, behavioral activation, and sleep disturbance. However, SSRIs may also contribute to behavioral activation or emotional blunting in some individuals, highlighting the importance of careful dose titration and monitoring.

## 3. Antipsychotics

Target symptoms: irritability, aggression, tantrums, self-injurious behavior.

### 3.1. Historical and Mechanistic Context

Antipsychotics have been used in ASD since the 1980s, when haloperidol demonstrated efficacy for reducing behavioral disturbances in children [47]. However, the risk of extrapyramidal side effects limited long-term use. The development of atypical antipsychotics provided better tolerability and became the mainstay for pharmacological management of severe irritability. These effects are thought to stem from modulation of D2 and 5-HT2A receptor pathways, which influence emotional reactivity and behavioral regulation through reward and limbic circuitry.

### 3.2. FDA-Approved Atypical Antipsychotics: Risperidone and Aripiprazole

Risperidone and aripiprazole are the only medications approved by the U.S. Food and Drug Administration (FDA) for irritability in children and adolescents with ASD. Double-blind randomized controlled trials demonstrated significant improvements in irritability, tantrums, and self-injury [20,21]. Systematic reviews and meta-analyses have confirmed these effects, with risperidone and aripiprazole consistently associated with large effect sizes in reducing irritability [22,23]. Multiple randomized controlled trials with samples typically ranging from 50 to over 200 participants have demonstrated significant reductions in irritability scores when compared with placebo, supporting their short-term efficacy.

### 3.3. Other Atypical Antipsychotics

However, evidence for other atypical antipsychotics such as olanzapine, quetiapine, and ziprasidone is limited to small or open-label studies, with less consistent efficacy and greater metabolic or sedative burden. Olanzapine has been studied in small case series, with some improvements in aggression but frequent weight gain [48]. Quetiapine has demonstrated benefit in open-label studies but also caused sedation and metabolic side effects [49]. Ziprasidone showed some efficacy in retrospective naturalistic studies, with the advantage of less weight gain but potential cardiac risks [50]. Paliperidone, the active metabolite of risperidone, has demonstrated benefit in adolescents and young adults in small open-label studies [51].

Clozapine remains a treatment of last resort for severe, refractory irritability and aggression in ASD. A recent scoping review described potential benefits but emphasized the need for careful monitoring of agranulocytosis and metabolic complications [36].

### 3.4. Safety Considerations

Atypical antipsychotics carry notable adverse effects. Weight gain and sedation are common, and long-term use may lead to metabolic syndrome, dyslipidemia, and insulin resistance [22]. Risperidone is also associated with hyperprolactinemia. Aripiprazole appears to have a more favorable metabolic profile, though akathisia and agitation are possible [21]. Guidelines recommend baseline and ongoing monitoring of weight, body mass index, fasting glucose, and lipid levels [35]. Weight gain and metabolic changes appear more pronounced with risperidone compared to aripiprazole, and current clinical guidelines recommend routine monitoring of weight, lipid profiles, and glucose during ongoing treatment [83].

### 3.5. Clinical Guideline Recommendations

The international guide to prescribing psychotropic medications for problem behaviors in intellectual disabilities supports antipsychotic use when severe aggression or self-injury threatens safety and behavioral interventions alone are insufficient [35]. The consensus across clinical guidelines is that atypical antipsychotics should be reserved for severe presentations, used at the lowest effective dose, and regularly re-evaluated for continued necessity [35]. Overall, while atypical antipsychotics provide reliable short-term reduction in irritability, their use remains primarily symptomatic, and ongoing monitoring and individualized treatment planning are necessary to balance benefits with metabolic and neurologic risks.

## 4. Stimulants

Target symptoms: hyperactivity, impulsivity, inattention, executive dysfunction.

### 4.1. Methylphenidate and Other Stimulants

Methylphenidate is the most widely studied stimulant in children with ASD. Early placebo-controlled crossover trials demonstrated moderate efficacy for hyperactivity, though adverse effects were more common than in typically developing children with ADHD [24]. The Cochrane systematic review by Sturman et al. synthesized randomized trials and confirmed reductions in hyperactivity but highlighted concerns about tolerability, including decreased appetite, insomnia, and increased irritability [54].

Amphetamine derivatives such as lisdexamfetamine have been evaluated in smaller studies. Case reports suggest potential benefits for comorbid ADHD symptoms but also note risk of affective lability and increased anxiety [55]. Broader pharmacological reviews, including work by Faraone et al., emphasize the need for individualized dosing strategies in neurodevelopmental populations given heightened sensitivity to side effects [56].

### 4.2. Modafinil and Novel Approaches

Modafinil, a wakefulness-promoting agent, has drawn attention for its potential anti-inflammatory and cognitive-enhancing properties. Preclinical studies demonstrate that modafinil reduces neuroinflammation and improves autism-like behaviors in animal models [57]. A medicinal chemistry review also highlighted structural modifications of modafinil derivatives with potential application in ASD [58]. While promising, clinical trials in humans are lacking, and modafinil is not currently recommended outside experimental contexts. Modafinil therefore remains investigational in ASD, with insufficient clinical evidence to support routine use.

## 5. Anticonvulsants

Target symptoms: seizures/epileptiform activity, irritability, aggression, mood instability.

### 5.1. Epilepsy and ASD

Epilepsy is a common comorbidity in ASD, with prevalence estimates ranging from 20 to 30% depending on cohort [17]. The overlap between epilepsy and behavioral dysregulation has prompted exploration of anticonvulsants as dual-purpose agents, addressing both seizures and behavioral symptoms. Anticonvulsants are frequently prescribed in ASD with comorbid epilepsy and have also been explored for behavioral regulation [84]. In addition to their use in seizure management, several anticonvulsants also function as mood stabilizers (thymostabilizers), modulating excitatory–inhibitory balance and affective regulation, which may be particularly relevant in ASD presentations marked by emotional lability or irritability [85].

### 5.2. Valproate

Valproate is one of the most frequently prescribed antiseizure medications in ASD. Preclinical studies of valproic acid (VPA) exposure have even been used to generate rodent models of autism, reflecting its mechanistic relevance [26]. In clinical contexts, valproate has been shown to reduce aggression and irritability in some patients, but teratogenicity, weight gain, and hepatotoxicity remain major concerns [27].

### 5.3. Lamotrigine

Lamotrigine, a sodium channel blocker with glutamate-modulating effects, has produced inconsistent findings in ASD. Belsito et al. conducted a randomized double-blind trial and found no significant benefit for behavioral symptoms compared to placebo. Nevertheless, some clinicians report improvements in mood instability, suggesting that lamotrigine may be useful in selecting subgroups [28]. Additionally, newer agents such as brivaracetam and cannabidiol are under investigation for ASD populations with comorbid epilepsy; however, current evidence remains preliminary, consisting primarily of small open-label and early-phase studies, and further controlled trials are needed to clarify their behavioral and seizure-related effects [86,87].

### 5.4. Levetiracetam

Levetiracetam is widely used for seizure control and has also been investigated for behavioral outcomes in ASD. Case reports suggest benefits in reducing aggression and self-injurious behavior [29], but other studies have raised concerns about behavioral activation and irritability [30]. A broader review highlighted that while levetiracetam is generally well-tolerated, close monitoring is necessary in individuals with ASD due to variable psychiatric side effects [31].

### 5.5. Topiramate and Other Antiseizure Drugs

Topiramate has been explored as an adjunctive treatment, particularly in combination with risperidone. Small studies suggest reductions in irritability, though cognitive side effects and sedation are limiting factors [32]. Frye et al. reviewed traditional and novel antiseizure medications in ASD and concluded that while seizure control remains paramount, evidence for behavioral benefits is mixed, necessitating larger controlled trials [18]. Evidence from multiple randomized controlled trials and meta-analyses supports improved tolerability and enhanced efficacy for topiramate used adjunctively with antipsychotics [88]. The synergistic mechanisms involve complementary actions on neurotransmitter systems and metabolic pathways [89].

**Part II. Emerging and Investigational Pharmacological Therapies** 

As understanding of ASD advances, a growing pipeline of investigational therapies aims to directly target the neurobiological mechanisms underlying social communication challenges, repetitive behaviors, mood dysregulation, and irritability. Unlike currently available medications—which primarily address co-occurring symptoms—emerging treatments focus on modulating specific molecular pathways implicated in ASD, including oxytocinergic and vasopressin signaling, glutamatergic transmission, neuroinflammation, mitochondrial and redox balance, and gene-level dysfunction (Table 2). Together, these emerging therapies highlight the shift toward mechanism-based, biologically stratified treatment strategies in ASD.

## 6. Neurotrophic, Oxidative Stress, and Immune-Modulating Agents

Target symptoms: irritability, repetitive behaviors, social deficits, immune dysregulation.

### 6.1. N-Acetylcysteine (NAC)

NAC, a glutathione precursor with antioxidant and glutamatergic-modulating properties, has emerged as one of the most promising adjunctive therapies in ASD. Randomized controlled pilot trials demonstrated that NAC reduced irritability and repetitive behaviors when compared with placebo [59]. Case reports further support its tolerability and potential utility in treatment-resistant patients [60]. Larger studies are warranted, but NAC’s favorable safety profile makes it an attractive candidate for adjunctive therapy.

### 6.2. Minocycline

Minocycline has gained attention as a potential treatment option for addressing neuroinflammation and microglial activation in autism spectrum disorder. ASD has been associated with elevated pro-inflammatory cytokines, increased microglial reactivity, and dysregulated immune signaling, suggesting that anti-inflammatory agents may have therapeutic value. Minocycline is a tetracycline-derived antibiotic with well-characterized neuroprotective effects, including the inhibition of microglial activation, reduction in oxidative stress, and modulation of inflammatory cascades such as TNF-α, IL-6, and NF-κB pathways. These mechanisms provide a plausible biological rationale for its use in ASD.

Preliminary clinical evidence supports this possibility. In a randomized, double-blind, placebo-controlled trial, minocycline used as an adjunct to risperidone led to improvements in irritability, hyperactivity, and social withdrawal in children with autistic disorder [61]. The study also reported an acceptable safety profile, although mild gastrointestinal symptoms and fatigue were observed. Despite promising findings, results remain limited by small sample sizes, short treatment durations, and heterogeneous outcome measures. Additional research is required to determine optimal dosing strategies, long-term safety, and whether ASD subgroups—such as those with elevated inflammatory markers—show greater benefit from minocycline therapy. Given the emerging connections between immune dysregulation and ASD pathophysiology, minocycline represents a mechanistically relevant drug candidate that may complement behavioral and pharmacological approaches already used in clinical practice.

### 6.3. Brain-Derived Neurotrophic Factor (BDNF) and Oxidative Stress

Altered BDNF levels have been implicated in ASD, linking neurotrophic signaling to abnormal neurodevelopment [62]. Reviews suggest that targeting BDNF pathways could improve synaptic plasticity and behavioral outcomes. Oxidative stress has also been repeatedly documented in ASD, with elevated markers of lipid peroxidation and reduced antioxidant capacity [9]. This has driven exploration of antioxidant therapies such as NAC, vitamin E, and omega-3 fatty acids, though evidence remains preliminary.

### 6.4. Immune and Inflammatory Mechanisms

Immune dysregulation is another proposed contributor to ASD pathophysiology. Celecoxib, a selective COX-2 inhibitor, demonstrated efficacy in reducing irritability when combined with risperidone in a randomized trial [95]. Other anti-inflammatory interventions, including minocycline and NAC, may partly exert their benefits through immunomodulatory effects.

Animal models further highlight the role of maternal immune activation in ASD-like phenotypes. Recent studies have demonstrated that nanoparticle-based therapies may prevent the transplacental passage of pathogenic maternal autoantibodies linked to autism risk, offering a novel preventive strategy [97]. Although still in preclinical stages, such approaches underscore the expanding horizon of immunologically targeted interventions.

### 6.5. Metabolic, Nutritional, and Microbiota-Directed Therapies

Emerging evidence indicates that a subset of individuals with ASD exhibit alterations in oxidative stress regulation, mitochondrial function, and one-carbon metabolism, as well as differences in gut microbiota composition and gastrointestinal physiology [9,11,12,18]. These findings have prompted investigation of metabolic and nutritional interventions as adjunctive strategies. Folate-related pathways are of particular interest: folinic acid and other folate formulations have shown preliminary benefits in selected subgroups characterized by folate receptor autoantibodies or methylation abnormalities, with some reports of improved language, adaptive functioning, and global behavior [11,15]. Vitamins B_6_ and B_12_ and other cofactors involved in neurotransmitter synthesis and redox balance have also been examined, although results remain mixed and often constrained by small sample sizes and heterogeneous study designs [9,11].

The gut–brain axis represents another promising therapeutic target. Children with ASD frequently experience gastrointestinal symptoms, and several studies have documented differences in microbiota composition and metabolite profiles compared with non-autistic peers [12,18]. Probiotic and prebiotic preparations, specialized diets, and other microbiota-directed interventions are being explored for their potential to influence behavior and emotional regulation through immunological, metabolic, and neuroendocrine pathways [12,18]. At present, evidence for these metabolic and microbiota-focused therapies remains preliminary, and no single protocol can be routinely recommended. Nevertheless, they illustrate how broader understanding of immune–metabolic contributions to ASD is generating novel avenues for pharmacological, nutraceutical, and dietary intervention that complement traditional psychotropic approaches [9,11,12,18].

### 6.6. Gene Therapy and Molecular Approaches

Advances in genetics and molecular neuroscience have also raised the possibility of targeted treatments for specific genetic forms of ASD, particularly syndromic presentations such as Rett syndrome, Fragile X syndrome, and Phelan-McDermid syndrome. In these conditions, well-characterized gene disruptions offer candidates for gene replacement or modulation. Experimental approaches include viral vector-mediated gene delivery, antisense oligonucleotides, RNA-targeted therapies, and CRISPR-based genome editing, with preclinical studies in relevant animal models demonstrating that partial restoration of gene function can improve synaptic physiology, neural circuit organization, and behavioral outcomes—even when interventions are introduced after early developmental windows [3,4].

Beyond monogenic syndromes, gene- and molecule-based strategies are being explored to influence broader neurodevelopmental pathways implicated in ASD, such as trophic factor signaling, synaptic scaffolding, and intracellular stress responses [10,11,12,62]. Although these approaches remain in early translational phases and raise important questions regarding safety, ethics, and equitable access, they represent a critical frontier in ASD therapeutics. In the longer term, integrating genomic, metabolomic, neuroimaging, and neuroimmune markers into clinical trials may allow pharmacologic and molecular interventions to be tailored to defined biological subgroups, moving the field toward precision medicine models of ASD care [2,3,5,6,8].

## 7. Glutamatergic Agents and NMDA Modulators

Target symptoms: social withdrawal, repetitive behaviors, irritability, cognitive rigidity.

### 7.1. Mechanistic Rationale

Abnormal glutamatergic signaling has been proposed as a core neurobiological feature of ASD, with studies demonstrating altered excitatory–inhibitory balance in cortical circuits [10]. Pharmacological interventions targeting glutamate release or NMDA receptor activity have therefore been investigated as potential therapies.

### 7.2. Riluzole

Riluzole, an agent that reduces presynaptic glutamate release, has demonstrated tolerability in clinical trials for mood and anxiety disorders. A systematic review and preliminary meta-analysis of riluzole in psychiatric conditions suggested potential benefit for mood stabilization and compulsive behaviors [63]. Although specific trials in ASD remain limited, riluzole’s mechanism positions it as a candidate for managing repetitive behaviors and emotional dysregulation.

### 7.3. Ketamine and NMDA Antagonists

Ketamine, a noncompetitive NMDA receptor antagonist, has gained attention for its rapid-acting antidepressant effects. Its potential in ASD has been explored in pilot studies and preclinical models. Systematic reviews suggest that NMDA antagonists, including ketamine, may attenuate irritability and social deficits, though the evidence base is still preliminary [64]. Preclinical and psychiatric evidence suggests NMDA blockade modulates glutamatergic signaling [64,65].

### 7.4. Broader NMDA-Targeting Strategies

Recent meta-analyses highlight that NMDA modulators as a class, including agents such as memantine and amantadine, warrant further study in ASD [64,66]. While small open-label studies have suggested improvements in hyperactivity and social responsiveness, placebo-controlled trials have yielded mixed results. At present, NMDA-targeting pharmacotherapies remain investigational in ASD, requiring larger and longer-term studies to establish efficacy and safety.

## 8. Adrenergic Agents (Clonidine and Guanfacine)

Target symptoms: hyperactivity, impulsivity, hyperarousal, anxiety, sleep problems.

### 8.1. Mechanistic Rationale

Adrenergic agents, particularly α2-adrenergic receptor agonists, are frequently used to manage hyperactivity and impulsivity in ASD, especially when stimulants are poorly tolerated. Their mechanism involves reducing presynaptic norepinephrine release, thereby calming overactive sympathetic responses [68]. The α_2_-adrenergic agonists reduce presynaptic norepinephrine release, leading to decreased hyperarousal and improved behavioral regulation [98]. Compared to stimulants, they may be better tolerated by individuals with ASD who exhibit comorbid tics, sleep disturbance, or pronounced irritability [99].

### 8.2. Clonidine

Clonidine was one of the earliest α2-agonists studied in autism. In small open-label trials and case reports, clonidine improved hyperactivity, sleep disturbance, and irritability [67]. However, sedation and hypotension were common, requiring careful dose titration. A systematic review confirmed clonidine’s modest efficacy, concluding that it may be most useful for children with prominent hyperarousal and sleep disturbance [53]. Clonidine, while supported primarily by case series and smaller open-label trials, appears useful for managing sleep disturbance and hyperactivity [52]. A systematic review by Banas and Sawchuk confirmed that clonidine can be effective for behavioral dysregulation in ASD but requires careful monitoring for hypotension and sedation [53].

### 8.3. Guanfacine

Compared to clonidine, guanfacine has a longer half-life and is less sedating. Controlled trials have demonstrated that guanfacine extended release significantly reduces hyperactivity and oppositional symptoms in ASD [69]. Clinical experience also supports guanfacine as a monotherapy or adjunct to stimulants for comorbid ADHD symptoms. Guanfacine has demonstrated efficacy in randomized controlled trials, improving hyperactivity and oppositional symptoms in children with ASD [25].

### 8.4. Clinical Considerations

Although α2-agonists are not FDA-approved for ASD, they are often used off-label in clinical practice. Their utility lies in cases where stimulants exacerbate anxiety, irritability, or sleep difficulties. Monitoring blood pressure and heart rate is recommended, particularly at higher doses [70]. Compared to stimulants, α_2_-agonists tend to produce smaller improvements in core attention but may be preferable in individuals with ASD who exhibit significant anxiety, tics, irritability, or sleep disturbance, as they are generally less activating [100]. Randomized trials of guanfacine extended-release typically include sample sizes of approximately 100–300 participants and demonstrate moderate reductions in hyperactivity and oppositional behaviors, with sedation and hypotension as the most common adverse effects [101]. In clinical practice, α_2_-agonists are often used when stimulants are poorly tolerated or when symptoms of hyperarousal and sleep disruption are prominent [102].

## 9. Discussion and Future Directions

This review expands the current pharmacotherapy literature in ASD by going beyond a summary of available guidelines and instead integrating cross-cutting biological mechanisms that underlie medication effects. Across drug classes, consistent patterns emerge involving disruptions in excitatory–inhibitory balance, chronic neuroinflammation, oxidative stress vulnerability, and altered neurotransmitter signaling. By drawing these mechanistic domains together, this review provides a more cohesive framework for understanding why certain medications benefit specific symptom clusters while others do not. Prior reviews have often examined these domains in isolation, whereas the present synthesis connects them into a broader system-level model that can guide more individualized and biologically informed treatment development [39,47,59]. This interpretive approach adds conceptual value beyond listing existing treatments and suggests clearer directions for future ASD pharmacotherapy research.

Several recent narrative and systematic reviews provide important context for the present synthesis. Aishworiya et al. offered an updated overview of psychopharmacological treatments in ASD, emphasizing principles of medication selection, dose titration, and the need to minimize unnecessary prescribing [94]. Davico et al. broadened the scope of pharmacological discussion to include a wide range of co-occurring conditions, such as tics, Tourette’s syndrome, and sleep disturbances, highlighting the clinical heterogeneity that clinicians must manage [93]. Manter et al. proposed guideline-oriented recommendations for managing common co-occurring psychiatric symptoms in ASD, underscoring the importance of individualized treatment plans, therapeutic drug monitoring, and structured follow-up [96]. Dell’Osso et al. synthesized available treatments ‘from old strategies to new options,’ integrating established psychotropics with emerging mechanism-based therapies grounded in neurobiological models of ASD [103]. The present review complements and extends these works by explicitly distinguishing medications currently used in routine clinical practice from emerging and investigational therapies, and by incorporating metabolic, microbiota-directed, and biotechnological approaches that reflect evolving etiopathogenetic concepts.

Pharmacological interventions in ASD address an array of behavioral and neurobiological targets, yet no single therapy effectively treats the full spectrum of symptoms. The strongest evidence continues to support atypical antipsychotics (risperidone, aripiprazole) for irritability [20,21,22,23], while SSRIs show mixed but sometimes meaningful benefits for comorbid anxiety and repetitive behaviors [41,42]. Stimulants and α2-agonists offer options for ADHD-like symptoms, but tolerability concerns necessitate careful monitoring [24,25,68]. Anticonvulsants remain essential for seizure management, though behavioral benefits are inconsistent [17,18,26,27,28,29,30,31,32]. Emerging approaches, including NAC, minocycline, riluzole, and NMDA modulators, highlight novel mechanistic targets but require further study [9,10,59,60,61,62,63,64,65,66,95,97]. These drug classes share several overlapping mechanistic pathways, including modulation of excitatory–inhibitory signaling, neurotransmitter network regulation, oxidative stress reduction, and neuroimmune interactions, suggesting that phenotypic symptoms in ASD arise from convergent neural circuit dysfunction rather than a single neurotransmitter abnormality.

### 9.1. Mechanistic Insights

Neuroimaging and neuropathological work suggest ASD involves widespread disruptions in cortical connectivity and synaptic signaling [5,6]. Studies of oxytocin receptors and peptide levels indicate that disruptions in the oxytocinergic system may contribute to social impairments, raising the possibility of oxytocin-based therapeutics [90,91]. Other mechanistic research points to microglial activation, oxidative stress, and excitatory–inhibitory imbalance, all of which inform emerging pharmacological strategies [9,10]. This cross-mechanistic convergence reinforces the rationale for developing biomarker-informed or circuit-level treatment approaches that align pharmacologic targets with underlying neural network dysregulation.

### 9.2. Safety and Long-Term Outcomes

Concerns about chronic antipsychotic exposure remain prominent. Nonhuman primate studies show that long-term antipsychotic treatment may alter brain volume and connectivity [92], highlighting the importance of balancing short-term behavioral gains against potential neurodevelopmental risks. Similarly, surveys of medication use emphasize that many children remain on psychotropic medications for extended periods without systematic re-evaluation [33,34].

### 9.3. Guidelines and Consensus

International guidelines underscore the principle that medications should target specific impairing symptoms, be initiated cautiously, and always be combined with behavioral interventions [35,38]. Recent systematic reviews also emphasize the importance of individualized medicine, including biomarker-guided treatment approaches, to optimize efficacy while minimizing adverse outcomes [2,3].

Across pharmacologic classes, one of the most consistent clinical challenges is the high prevalence of psychotropic polypharmacy in ASD, particularly among individuals with multiple psychiatric and neurological comorbidities [13,14,22,23,33,34,93,94,96,103]. Combinations of antipsychotics, stimulants, antidepressants, anticonvulsants, adrenergic agents, and adjunctive therapies increase the risk of pharmacodynamic and pharmacokinetic interactions, cumulative adverse effects, and long-term metabolic burden. Consensus guidelines therefore recommend that each medication be linked to clearly defined target symptoms, introduced one at a time, and prescribed at the lowest effective dose with planned intervals for re-evaluation and possible tapering [35,36,38,93,94,96]. Systematic monitoring of weight, body mass index, blood pressure, fasting glucose, lipid profiles, and, when indicated, serum drug levels and prolactin can help detect emerging toxicity and guide dose adjustments [22,23]. In addition, deprescribing strategies—such as stepwise reduction of agents with the least clear ongoing benefit—are increasingly recognized as essential components of ASD pharmacotherapy. Future work should develop and validate structured algorithms and decision aids to support clinicians and families in optimizing polypharmacy while balancing symptom control, tolerability, and long-term health outcomes.

### 9.4. Emerging and Preventive Approaches

Novel immune-modulating strategies, such as nanoparticle-based therapies to block maternal autoantibody transfer, illustrate the potential for preventive interventions [97]. Advances in genetics and metabolomics may allow clinicians to identify subgroups of individuals with ASD who are most likely to benefit from pharmacotherapies [11]. Trials of agents targeting neuroinflammation, oxidative stress, and glutamatergic signaling reflect a shift toward mechanism-based treatment development [4,12]. However, these approaches remain preliminary, and their translation into clinical interventions will require replication in larger, controlled human studies, with standardized outcome measures.

### 9.5. Future Research Priorities

Looking forward, clinical research should prioritize:

- Large, well-controlled trials of promising novel agents (e.g., NAC, riluzole, ketamine).
- Longitudinal safety studies, particularly for antipsychotics and stimulants used in children.
- Biomarker integration to predict treatment response (e.g., metabolomic profiling [44], oxytocin receptor expression [90]).
- Preventive strategies that target maternal immune activation and early neurodevelopmental pathways [97].
- Combination therapies that integrate behavioral interventions, psychopharmacology, and family support, reflecting the multifaceted nature of ASD.

Ultimately, while substantial progress has been made, achieving personalized, mechanism-driven pharmacotherapy remains the central challenge and opportunity in ASD treatment. In clinical practice, medication selection in ASD often requires balancing symptom targets with individual profiles of comorbidity, developmental stage, and tolerability. For example, atypical antipsychotics are most effective for severe irritability, whereas SSRIs may be more appropriate for anxiety or obsessive–compulsive features, and stimulants or α_2_-adrenergic agonists may be useful for attentional dysregulation or hyperarousal. However, the presence of overlapping symptoms across individuals highlights the need for treatment algorithms that integrate symptom clusters rather than isolated diagnostic categories. Future research should therefore prioritize identifying which medication classes are most effective for specific symptom dimensions and subgroup profiles, as well as developing evidence-informed combination strategies for cases involving multiple comorbid symptoms.

## 10. Conclusions

Pharmacological treatments for ASD have advanced considerably, yet the therapeutic landscape remains fragmented and symptom-targeted rather than curative. Atypical antipsychotics provide robust evidence for managing irritability, while SSRIs, stimulants, anticonvulsants, and adrenergic agents offer variable benefits for comorbid symptoms. Emerging pharmacotherapies that target oxidative stress, neuroinflammation, and glutamatergic dysregulation hold promise but remain preliminary, with limited replication in large, controlled clinical trials.

At the same time, the current evidence base for pharmacologic interventions in ASD has important limitations. Many agents have been evaluated in relatively small, short-duration trials with heterogeneous samples and outcome measures, making it difficult to compare results across studies or to draw firm conclusions about long-term effectiveness and safety [9,18,22,23]. Longitudinal data on metabolic, neurologic, and cognitive outcomes for children and adolescents receiving chronic antipsychotic, stimulants, or combination regimens remain sparse [22,33,34,92]. Few trials incorporate biomarker-based stratification to identify subgroups most likely to benefit from particular mechanisms of action, despite growing evidence of biological heterogeneity in ASD [2,3,5,7,8,11,12]. Future efforts should prioritize adequately powered, rigorously controlled trials of promising agents, standardized and clinically meaningful outcome measures, and long-term follow-up designs that capture developmental trajectories. Integrating genomic, metabolomic, neuroimaging, and neuroimmune markers into clinical trials may support more precise, mechanism-driven prescribing and help move ASD care toward genuinely personalized pharmacotherapy embedded within comprehensive behavioral and educational frameworks [2,3,5,6,7,8,11,12].

The heterogeneity of ASD underscores the need for precision medicine approaches that incorporate biomarkers, genetic profiling, and individualized treatment algorithms to match specific neurobiological mechanisms with responsive subgroups. Future research must also prioritize long-term safety and developmental outcomes, particularly given concerns regarding metabolic effects and potential neurodevelopmental impacts of chronic psychotropic exposure in children and adolescents. Ultimately, pharmacotherapy in ASD is most effective when integrated with behavioral and educational interventions rather than used as a stand-alone strategy. Moving toward comprehensive, multimodal treatment frameworks that align mechanistic targets with individual clinical profiles represents the key challenge and opportunity for advancing care in ASD.

## Figures and Tables

**Table 1:** Mechanisms and pathways in pharmacological treatments for ASD.

Drug Class	Target Symptoms	Drugs	Mechanism	Pathways/Targets	Supporting References
SSRIs (Selective Serotonin Reuptake Inhibitors)	Anxiety, obsessive–compulsive behaviors	*Fluoxetine, Sertraline*	Inhibit serotonin reuptake, increasing serotonin availability in the synapse	Serotonin transporter (SERT), 5-HT signaling	[37,39,40,41,42,43,44,45,46]
Antipsychotics	Irritability, aggression, tantrums	*Risperidone, Aripiprazole*	Block D_2_ and 5-HT_2_A receptors to regulate dopamine and serotonin signaling	Dopamine D_2_, serotonin 5-HT_2_A receptors	[20,21,22,23,35,36,47,48,49,50,51]
Stimulants	ADHD-like symptoms (inattention, impulsivity, hyperactivity)	*Methylphenidate, Amphetamine, Modafinil, Lisdexamfetamine, Guanfacine*	Increase dopamine and norepinephrine by inhibiting reuptake; modafinil also reduces neuroinflammation and enhances arousal	Dopamine transporter (DAT), norepinephrine transporter (NET), glutamatergic excitotoxicity (for modafinil)	[24,25,52,53,54,55,56,57,58]
Anticonvulsants	Seizures, aggression, mood lability	*Valproic acid, Lamotrigine, Topiramate*	Inhibit Na^+^/Ca^2+^ channels, increase GABA activity	Sodium channels (Na^+^), calcium channels (Ca^2+^), GABA receptors	[17,18,26,27,28,29,30,31,32]
Neurotrophic and Anti-inflammatory Agents	Brain connectivity, neuroinflammation	*Minocycline, N-acetylcysteine (NAC), Baclofen*	Modulate microglial activation and oxidative stress; influence BDNF pathways	Microglia, BDNF signaling, oxidative stress markers	[9,59,60,61,62]
NMDA Modulators	Rigidity, repetitive behaviors	*Riluzole*	Reduce glutamatergic excitotoxicity by inhibiting glutamate release and receptor activity	NMDA receptors, glutamatergic pathways	[10,63,64,65,66]
α_2_-Adrenergic Agonists	Hyperactivity, sleep disturbance, aggression	*Clonidine, Guanfacine*	Decrease norepinephrine release, calming CNS hyperarousal	α_2_-adrenergic receptors (α_2_)	[25,52,53,67,68,69,70]
Sleep Agents	Sleep-onset insomnia, nighttime awakenings	*Melatonin*	Regulates circadian rhythms through MT1 and MT2 receptor activation, promoting physiological sleep initiation	Melatonin receptors (MT1/MT2), circadian timing pathways	[18]
Sedating Antidepressants	Insomnia with co-occurring anxiety, mood dysregulation, or hyperarousal	*Trazodone, Mirtazapine*	Trazodone: Serotonin antagonist and reuptake inhibitor with sedative properties Mirtazapine: Noradrenergic and specific serotonergic modulator with potent antihistaminergic activity	5-HT2A/5-HT2C receptors, histamine H1 receptors, noradrenergic pathways	[52,53,67]
Appetite-Stimulating/Feeding Interventions	Low appetite, poor weight gain, feeding selectivity	*Cyproheptadine*	Antihistamine with antiserotonergic effects that enhance appetite and feeding behavior	Histamine H1 receptor blockade; serotonin receptor antagonism	[18]

**Table 2:** Emerging and investigational pharmacological and biotechnological interventions in ASD.

Therapy	Proposed Mechanism	Primary Targets/Rationale:	Supporting References
Oxytocin and Oxytocinergic Modulators	Enhances social salience, reward processing, and affiliative behavior via oxytocin receptor signaling	Social communication, social motivation, emotional reciprocity	[33,90,91,92]
Vasopressin Receptor Agents	Modulates social behavior, stress responses, and emotional regulation via V1A receptor pathways	Social behavior, anxiety-related symptoms, irritability	[54,92]
N-Acetylcysteine (NAC)	Antioxidant activity, glutathione enhancement, and modulation of glutamatergic transmission	Irritability, repetitive behaviors, oxidative stress imbalance	[9,59,60,93]
Minocycline and Other Anti-inflammatory Agents	Reduces microglial activation; decreases neuroinflammation and oxidative stress	Irritability, neuroinflammation-driven behavioral symptoms	[61,94]
Riluzole	Reduces presynaptic glutamate release; enhances glutamate clearance	Cognitive rigidity, repetitive behaviors, mood dysregulation	[64]
NMDA Receptor Modulators (e.g., Memantine, Ketamine-like agents)	Modulates NMDA receptor signaling to improve synaptic plasticity and reduce excitotoxicity	Repetitive behaviors, cognitive inflexibility, treatment-resistant irritability	[64,65,66]
COX-2 Inhibitors (e.g., Celecoxib)	Attenuates neuroinflammation by inhibiting cyclooxygenase-2 pathways	Adjunctive reduction of irritability, aggression, and inflammatory-driven behavior	[94,95]
Microbiota-Directed Therapies (Probiotics, Prebiotics, Diet-based interventions)	Modulates gut–brain axis, immune–metabolic signaling, and microbial metabolite production	Gastrointestinal dysfunction, behavioral regulation, mood stability	[18,94]
Metabolic and One-Carbon Pathway Supports (e.g., Folinic Acid)	Targets methylation cycles, oxidative stress pathways, and folate receptor dysfunction	Language development, adaptive function, global behavior in biologically defined subgroups	[9,18,93]
Gene and Molecular Therapies	Gene replacement, antisense oligonucleotides, RNA-targeted therapeutics, CRISPR-based editing	Syndromic ASD variants (Rett, Fragile X, Phelan–McDermid), severe neurodevelopmental impairment	[3,8,93,96]

## Data Availability

No new data were created or analyzed in this study.

## References

[1] Maenner M.J., Shaw K.A., Bak J., Washington A., Patrick M., DiRienzo M., Christensen D.L., Wiggins L.D., Pettygrove S., Andrews J.G. (2020). Prevalence of Autism Spectrum Disorder Among Children Aged 8 Years—Autism and Developmental Disabilities Monitoring Network, 11 Sites, United States, 2016. MMWR Surveill. Summ..

[2] Lai M.C., Lombardo M.V., Baron-Cohen S. (2014). Autism. Lancet.

[3] Lord C., Elsabbagh M., Baird G., Veenstra-Vanderweele J. (2018). Autism spectrum disorder. Lancet.

[4] Sharma S.R., Gonda X., Tarazi F.I. (2018). Autism Spectrum Disorder: Classification, diagnosis and therapy. Pharmacol. Ther..

[5] Ecker C., Bookheimer S.Y., Murphy D.G. (2015). Neuroimaging in autism spectrum disorder: Brain structure and function across the lifespan. Lancet Neurol..

[6] Courchesne E., Campbell K., Solso S. (2011). Brain growth across the life span in autism: Age-specific changes in anatomical pathology. Brain Res..

[7] Kolevzon A., Gross R., Reichenberg A. (2007). Prenatal and perinatal risk factors for autism: A review and integration of findings. Arch. Pediatr. Adolesc. Med..

[8] Sandin S., Lichtenstein P., Kuja-Halkola R., Larsson H., Hultman C.M., Reichenberg A. (2014). The familial risk of autism. JAMA.

[9] Chauhan A., Chauhan V. (2006). Oxidative stress in autism. Pathophysiology.

[10] Uzunova G., Pallanti S., Hollander E. (2016). Excitatory/inhibitory imbalance in autism spectrum disorders: Implications for interventions and therapeutics. World J. Biol. Psychiatry.

[11] Rose S., Frye R.E., Slattery J., Wynne R., Tippett M., Pavliv O., Melnyk S., James S.J. (2014). Oxidative stress induces mitochondrial dysfunction in a subset of autism lymphoblastoid cell lines in a well-matched case control cohort. PLoS ONE.

[12] Matta S.M., Hill-Yardin E.L., Crack P.J. (2019). The influence of neuroinflammation in Autism Spectrum Disorder. Brain Behav. Immun..

[13] Jobski K., Höfer J., Hoffmann F., Bachmann C. (2017). Use of psychotropic drugs in patients with autism spectrum disorders: A systematic review. Acta Psychiat. Scand..

[14] Coleman D.M., Adams J.B., Anderson A.L., Frye R.E. (2019). Rating of the Effectiveness of 26 Psychiatric and Seizure Medications for Autism Spectrum Disorder: Results of a National Survey. J. Child Adolesc. Psychopharmacol..

[15] Vasa R.A., Carroll L.M., Nozzolillo A.A., Mahajan R., Mazurek M.O., Bennett A.E., Wink L.K., Bernal M.P. (2014). A systematic review of treatments for anxiety in youth with autism spectrum disorders. J. Autism Dev. Disord..

[16] Deb S., Akrout Brizard B., Limbu B. (2020). Association between epilepsy and challenging behaviour in adults with intellectual disabilities: Systematic review and meta-analysis. BJPsych Open.

[17] Tuchman R., Rapin I. (2002). Epilepsy in autism. Lancet Neurol..

[18] Frye R.E., Rossignol D., Casanova M.F., Brown G.L., Martin V., Edelson S., Coben R., Lewine J., Slattery J.C., Lau C. (2013). A review of traditional and novel treatments for seizures in autism spectrum disorder: Findings from a systematic review and expert panel. Front. Public Health.

[19] Vohra R., Madhavan S., Sambamoorthi U., St Peter C. (2014). Access to services, quality of care, and family impact for children with autism, other developmental disabilities, and other mental health conditions. Autism.

[20] McDougle C.J., Scahill L., Aman M.G., McCracken J.T., Tierney E., Davies M., Arnold L.E., Posey D.J., Martin A., Ghuman J.K. (2005). Risperidone for the core symptom domains of autism: Results from the study by the autism network of the research units on pediatric psychopharmacology. Am. J. Psychiatry.

[21] Marcus R.N., Owen R., Kamen L., Manos G., McQuade R.D., Carson W.H., Aman M.G. (2009). A placebo-controlled, fixed-dose study of aripiprazole in children and adolescents with irritability associated with autistic disorder. J. Am. Acad. Child Adolesc. Psychiatry.

[22] D’Alo G.L., De Crescenzo F., Amato L., Cruciani F., Davoli M., Fulceri F., Minozzi S., Mitrova Z., Morgano G.P., Nardocci F. (2021). Impact of antipsychotics in children and adolescents with autism spectrum disorder: A systematic review and meta-analysis. Health Qual. Life Outcomes.

[23] Fung L.K., Mahajan R., Nozzolillo A., Bernal P., Krasner A., Jo B., Coury D., Whitaker A., Veenstra-Vanderweele J., Hardan A.Y. (2016). Pharmacologic Treatment of Severe Irritability and Problem Behaviors in Autism: A Systematic Review and Meta-analysis. Pediatrics.

[24] Handen B.L., Johnson C.R., Lubetsky M. (2000). Efficacy of methylphenidate among children with autism and symptoms of attention-deficit hyperactivity disorder. J. Autism Dev. Disord..

[25] Scahill L., Aman M.G., McDougle C.J., McCracken J.T., Tierney E., Dziura J., Arnold L.E., Posey D., Young C., Shah B. (2006). A prospective open trial of guanfacine in children with pervasive developmental disorders. J. Child Adolesc. Psychopharmacol..

[26] Chomiak T., Turner N., Hu B. (2013). What We Have Learned about Autism Spectrum Disorder from Valproic Acid. Patholog. Res. Int..

[27] Hollander E., Soorya L., Wasserman S., Esposito K., Chaplin W., Anagnostou E. (2006). Divalproex sodium vs. placebo in the treatment of repetitive behaviours in autism spectrum disorder. Int. J. Neuropsychopharmacol..

[28] Belsito K.M., Law P.A., Kirk K.S., Landa R.J., Zimmerman A.W. (2001). Lamotrigine therapy for autistic disorder: A randomized, double-blind, placebo-controlled trial. J. Autism Dev. Disord..

[29] Deriaz N., Willi J.P., Orihuela-Flores M., Galli Carminati G., Ratib O. (2012). Treatment with levetiracetam in a patient with pervasive developmental disorders, severe intellectual disability, self-injurious behavior, and seizures: A case report. Neurocase.

[30] Farooq M.U., Bhatt A., Majid A., Gupta R., Khasnis A., Kassab M.Y. (2009). Levetiracetam for managing neurologic and psychiatric disorders. Am. J. Health Syst. Pharm..

[31] Halma E., de Louw A.J., Klinkenberg S., Aldenkamp A.P., DM I.J., Majoie M. (2014). Behavioral side-effects of levetiracetam in children with epilepsy: A systematic review. Seizure.

[32] Rezaei V., Mohammadi M.R., Ghanizadeh A., Sahraian A., Tabrizi M., Rezazadeh S.A., Akhondzadeh S. (2010). Double-blind, placebo-controlled trial of risperidone plus topiramate in children with autistic disorder. Prog. Neuropsychopharmacol. Biol. Psychiatry.

[33] Mandell D.S., Morales K.H., Marcus S.C., Stahmer A.C., Doshi J., Polsky D.E. (2008). Psychotropic medication use among Medicaid-enrolled children with autism spectrum disorders. Pediatrics.

[34] Murray M.L., Hsia Y., Glaser K., Simonoff E., Murphy D.G., Asherson P.J., Eklund H., Wong I.C. (2014). Pharmacological treatments prescribed to people with autism spectrum disorder (ASD) in primary health care. Psychopharmacology.

[35] Deb S., Kwok H., Bertelli M., Salvador-Carulla L., Bradley E., Torr J., Barnhill J., Guideline Development Group of the WPA Section on Psychiatry of Intellectual Disability (2009). International guide to prescribing psychotropic medication for the management of problem behaviours in adults with intellectual disabilities. World Psychiatry.

[36] da Rosa A., Bezerra O.S., Rohde L.A., Graeff-Martins A.S. (2024). Exploring clozapine use in severe psychiatric symptoms associated with autism spectrum disorder: A scoping review. J. Psychopharmacol..

[37] Wood J.J., Drahota A., Sze K., Har K., Chiu A., Langer D.A. (2009). Cognitive behavioral therapy for anxiety in children with autism spectrum disorders: A randomized, controlled trial. J. Child Psychol. Psychiatry.

[38] Crowe B.H., Salt A.T. (2015). Autism: The management and support of children and young people on the autism spectrum (NICE Clinical Guideline 170). Arch. Dis. Child. Educ. Pract. Ed..

[39] Chugani D.C. (2002). Role of altered brain serotonin mechanisms in autism. Mol. Psychiatry.

[40] Muller C.L., Anacker A.M.J., Veenstra-VanderWeele J. (2016). The serotonin system in autism spectrum disorder: From biomarker to animal models. Neuroscience.

[41] Williams K., Brignell A., Randall M., Silove N., Hazell P. (2013). Selective serotonin reuptake inhibitors (SSRIs) for autism spectrum disorders (ASD). Cochrane Database Syst. Rev..

[42] Reddihough D.S., Marraffa C., Mouti A., O’Sullivan M., Lee K.J., Orsini F., Hazell P., Granich J., Whitehouse A.J.O., Wray J. (2019). Effect of Fluoxetine on Obsessive-Compulsive Behaviors in Children and Adolescents with Autism Spectrum Disorders: A Randomized Clinical Trial. JAMA.

[43] Golub M.S., Hogrefe C.E., Bulleri A.M. (2016). Peer social interaction is facilitated in juvenile rhesus monkeys treated with fluoxetine. Neuropharmacology.

[44] He Y., Hogrefe C.E., Grapov D., Palazoglu M., Fiehn O., Turck C.W., Golub M.S. (2014). Identifying individual differences of fluoxetine response in juvenile rhesus monkeys by metabolite profiling. Transl. Psychiatry.

[45] King B.H., Hollander E., Sikich L., McCracken J.T., Scahill L., Bregman J.D., Donnelly C.L., Anagnostou E., Dukes K., Sullivan L. (2009). Lack of efficacy of citalopram in children with autism spectrum disorders and high levels of repetitive behavior: Citalopram ineffective in children with autism. Arch. Gen. Psychiatry.

[46] Potter L.A., Scholze D.A., Biag H.M.B., Schneider A., Chen Y., Nguyen D.V., Rajaratnam A., Rivera S.M., Dwyer P.S., Tassone F. (2019). A Randomized Controlled Trial of Sertraline in Young Children with Autism Spectrum Disorder. Front. Psychiatry.

[47] Anderson L.T., Campbell M., Grega D.M., Perry R., Small A.M., Green W.H. (1984). Haloperidol in the treatment of infantile autism: Effects on learning and behavioral symptoms. Am. J. Psychiatry.

[48] Stavrakaki C., Antochi R., Emery P.C. (2004). Olanzapine in the treatment of pervasive developmental disorders: A case series analysis. J. Psychiatry Neurosci..

[49] Golubchik P., Sever J., Weizman A. (2011). Low-dose quetiapine for adolescents with autistic spectrum disorder and aggressive behavior: Open-label trial. Clin. Neuropharmacol..

[50] Dominick K., Wink L.K., McDougle C.J., Erickson C.A. (2015). A Retrospective Naturalistic Study of Ziprasidone for Irritability in Youth with Autism Spectrum Disorder. J. Child Adolesc. Psychopharmacol..

[51] Stigler K.A., Mullett J.E., Erickson C.A., Posey D.J., McDougle C.J. (2012). Paliperidone for irritability in adolescents and young adults with autistic disorder. Psychopharmacology.

[52] Jaselskis C.A., Cook E.H., Fletcher K.E., Leventhal B.L. (1992). Clonidine treatment of hyperactive and impulsive children with autistic disorder. J. Clin. Psychopharmacol..

[53] Banas K., Sawchuk B. (2020). Clonidine as a Treatment of Behavioural Disturbances in Autism Spectrum Disorder: A Systematic Literature Review. J. Can. Acad. Child Adolesc. Psychiatry.

[54] Sturman N., Deckx L., van Driel M.L. (2017). Methylphenidate for children and adolescents with autism spectrum disorder. Cochrane Database Syst. Rev..

[55] Calderon P.D., de la Puente A.I., Moreno M.G., Fernandez R.F. (2023). Lisdexamfetamine in combination with guanfacine as an effective treatment in the management of behavioral disturbances in patients with Attention Deficit Hyperactivity Disorder (ADHD) and Autism Spectrum Disorder (ASD). Case report. Eur. Psychiat..

[56] Faraone S.V. (2018). The pharmacology of amphetamine and methylphenidate: Relevance to the neurobiology of attention-deficit/hyperactivity disorder and other psychiatric comorbidities. Neurosci. Biobehav. Rev..

[57] Bagcioglu E., Solmaz V., Erbas O., Ozkul B., Cakar B., Uyanikgil Y., Sogut I. (2023). Modafinil Improves Autism-like Behavior in Rats by Reducing Neuroinflammation. J. Neuroimmune Pharmacol..

[58] Gerrard P., Malcolm R. (2007). Mechanisms of modafinil: A review of current research. Neuropsychiatr. Dis. Treat..

[59] Hardan A.Y., Fung L.K., Libove R.A., Obukhanych T.V., Nair S., Herzenberg L.A., Frazier T.W., Tirouvanziam R. (2012). A randomized controlled pilot trial of oral N-acetylcysteine in children with autism. Biol. Psychiatry.

[60] Ghanizadeh A., Derakhshan N. (2012). N-acetylcysteine for treatment of autism, a case report. J. Res. Med. Sci..

[61] Ghaleiha A., Alikhani R., Kazemi M.R., Mohammadi M.R., Mohammadinejad P., Zeinoddini A., Hamedi M., Shahriari M., Keshavarzi Z., Akhondzadeh S. (2016). Minocycline as Adjunctive Treatment to Risperidone in Children with Autistic Disorder: A Randomized, Double-Blind Placebo-Controlled Trial. J. Child Adolesc. Psychopharmacol..

[62] Zheng Z., Zhang L., Zhu T., Huang J., Qu Y., Mu D. (2016). Peripheral brain-derived neurotrophic factor in autism spectrum disorder: A systematic review and meta-analysis. Sci. Rep..

[63] de Boer J.N., Vingerhoets C., Hirdes M., McAlonan G.M., Amelsvoort T.V., Zinkstok J.R. (2019). Efficacy and tolerability of riluzole in psychiatric disorders: A systematic review and preliminary meta-analysis. Psychiatry Res..

[64] Dessus-Gilbert M.L., Nourredine M., Zimmer L., Rolland B., Geoffray M.M., Auffret M., Jurek L. (2024). NMDA antagonist agents for the treatment of symptoms in autism spectrum disorder: A systematic review and meta-analysis. Front. Pharmacol..

[65] Qin X.Y., Feng J.C., Cao C., Wu H.T., Loh Y.P., Cheng Y. (2016). Association of Peripheral Blood Levels of Brain-Derived Neurotrophic Factor with Autism Spectrum Disorder in Children: A Systematic Review and Meta-analysis. JAMA Pediatr..

[66] Erickson C.A., Posey D.J., Stigler K.A., Mullett J., Katschke A.R., McDougle C.J. (2007). A retrospective study of memantine in children and adolescents with pervasive developmental disorders. Psychopharmacology.

[67] Ming X., Gordon E., Kang N., Wagner G.C. (2008). Use of clonidine in children with autism spectrum disorders. Brain Dev..

[68] Scahill L., McCracken J.T., King B.H., Rockhill C., Shah B., Politte L., Sanders R., Minjarez M., Cowen J., Mullett J. (2015). Extended-Release Guanfacine for Hyperactivity in Children with Autism Spectrum Disorder. Am. J. Psychiatry.

[69] Charman T. (2018). Mapping Early Symptom Trajectories in Autism Spectrum Disorder: Lessons and Challenges for Clinical Practice and Science. J. Am. Acad. Child Adolesc. Psychiatry.

[70] Posey D.J., Puntney J.I., Sasher T.M., Kem D.L., McDougle C.J. (2004). Guanfacine treatment of hyperactivity and inattention in pervasive developmental disorders: A retrospective analysis of 80 cases. J. Child Adolesc. Psychopharmacol..

[71] Albert P.R. (2012). Transcriptional regulation of the 5-HT1A receptor: Implications for mental illness. Philos. Trans. R. Soc. B Biol. Sci.

[72] Blier P. (2001). Crosstalk between the norepinephrine and serotonin systems and its role in the antidepressant response. J. Psychiatry Neurosci..

[73] David S.P., Murthy N.V., Rabiner E.A., Munafo M.R., Johnstone E.C., Jacob R., Walton R.T., Grasby P.M. (2005). A functional genetic variation of the serotonin (5-HT) transporter affects 5-HT1A receptor binding in humans. J. Neurosci..

[74] Pawlowski T., Malyszczak K., Pawlak D., Inglot M., Zalewska M., Grzywacz A., Radkowski M., Laskus T., Janocha-Litwin J., Frydecka D. (2023). HTR1A, TPH2, and 5-HTTLPR Polymorphisms and Their Impact on the Severity of Depressive Symptoms and on the Concentration of Tryptophan Catabolites during Hepatitis C Treatment with Pegylated Interferon-alpha2a and Oral Ribavirin (PEG-IFN-alpha2a/RBV). Cells.

[75] Bauman M.D., Schumann C.M. (2018). Advances in nonhuman primate models of autism: Integrating neuroscience and behavior. Exp. Neurol..

[76] Watson K.K., Ghodasra J.H., Platt M.L. (2009). Serotonin transporter genotype modulates social reward and punishment in rhesus macaques. PLoS ONE.

[77] Baudat M., de Kort A.R., van den Hove D.L.A., Joosten E.A. (2022). Early-life exposure to selective serotonin reuptake inhibitors: Long-term effects on pain and affective comorbidities. Eur. J. Neurosci..

[78] Zanni G., van Dijk M.T., Cagliostro M.C., Sepulveda P., Pini N., Rose A.L., Kesin A.L., Lugo-Candelas C., Goncalves P.D., MacKay A.S. (2025). Perinatal SSRI exposure impacts innate fear circuit activation and behavior in mice and humans. Nat. Commun..

[79] Strawn J.R., Mills J.A., Schroeder H., Mossman S.A., Varney S.T., Ramsey L.B., Poweleit E.A., Desta Z., Cecil K., DelBello M.P. (2020). Escitalopram in Adolescents with Generalized Anxiety Disorder: A Double-Blind, Randomized, Placebo-Controlled Study. J. Clin. Psychiatry.

[80] Strawn J.R., Poweleit E.A., Mills J.A., Schroeder H.K., Neptune Z.A., Specht A.M., Farrow J.E., Zhang X., Martin L.J., Ramsey L.B. (2021). Pharmacogenetically Guided Escitalopram Treatment for Pediatric Anxiety Disorders: Protocol for a Double-Blind Randomized Trial. J. Pers. Med..

[81] Rink L., Adams A., Braun C., Bschor T., Kuhr K., Baethge C. (2022). Dose-Response Relationship in Selective Serotonin and Norepinephrine Reuptake Inhibitors in the Treatment of Major Depressive Disorder: A Meta-Analysis and Network Meta-Analysis of Randomized Controlled Trials. Psychother. Psychosom..

[82] Weiss M.D., Wasdell M.B., Bomben M.M., Rea K.J., Freeman R.D. (2006). Sleep hygiene and melatonin treatment for children and adolescents with ADHD and initial insomnia. J. Am. Acad. Child Adolesc. Psychiatry.

[83] Robinson D.G., Gallego J.A., John M., Petrides G., Hassoun Y., Zhang J.P., Lopez L., Braga R.J., Sevy S.M., Addington J. (2015). A Randomized Comparison of Aripiprazole and Risperidone for the Acute Treatment of First-Episode Schizophrenia and Related Disorders: 3-Month Outcomes. Schizophr. Bull..

[84] Hirota T., Veenstra-Vanderweele J., Hollander E., Kishi T. (2014). Antiepileptic medications in autism spectrum disorder: A systematic review and meta-analysis. J. Autism Dev. Disord..

[85] Davico C., Canavese C., Vittorini R., Gandione M., Vitiello B. (2018). Anticonvulsants for Psychiatric Disorders in Children and Adolescents: A Systematic Review of Their Efficacy. Front. Psychiatry.

[86] Jawed B., Esposito J.E., Pulcini R., Zakir S.K., Botteghi M., Gaudio F., Savio D., Martinotti C., Martinotti S., Toniato E. (2024). The Evolving Role of Cannabidiol-Rich Cannabis in People with Autism Spectrum Disorder: A Systematic Review. Int. J. Mol. Sci..

[87] Hassan M.A., Awad A.A., Marey A., Amin A.M., Elshahat A., El-Moslemani M., Mansour A., Ramadan S., Aldemerdash M.A. (2025). Efficacy and safety of Brivaracetam as adjunctive therapy in pediatric epilepsy: A systematic review and meta-analysis. Neurol. Sci..

[88] Zheng W., Xiang Y.T., Xiang Y.Q., Li X.B., Ungvari G.S., Chiu H.F., Correll C.U. (2016). Efficacy and safety of adjunctive topiramate for schizophrenia: A meta-analysis of randomized controlled trials. Acta Psychiatr. Scand..

[89] Okuyama Y., Oya K., Matsunaga S., Kishi T., Iwata N. (2016). Efficacy and tolerability of topiramate-augmentation therapy for schizophrenia: A systematic review and meta-analysis of randomized controlled trials. Neuropsychiatr. Dis. Treat..

[90] Loth E., Poline J.B., Thyreau B., Jia T., Tao C., Lourdusamy A., Stacey D., Cattrell A., Desrivieres S., Ruggeri B. (2014). Oxytocin receptor genotype modulates ventral striatal activity to social cues and response to stressful life events. Biol. Psychiatry.

[91] Zhang H.F., Dai Y.C., Wu J., Jia M.X., Zhang J.S., Shou X.J., Han S.P., Zhang R., Han J.S. (2016). Plasma Oxytocin and Arginine-Vasopressin Levels in Children with Autism Spectrum Disorder in China: Associations with Symptoms. Neurosci. Bull..

[92] Konopaske G.T., Dorph-Petersen K.A., Pierri J.N., Wu Q., Sampson A.R., Lewis D.A. (2007). Effect of chronic exposure to antipsychotic medication on cell numbers in the parietal cortex of macaque monkeys. Neuropsychopharmacology.

[93] Davico C., Secci I., Vendrametto V., Vitiello B. (2023). Pharmacological treatments in autism spectrum disorder: A narrative review. J. Psychopathol..

[94] Aishworiya R., Valica T., Hagerman R., Restrepo B. (2022). An Update on Psychopharmacological Treatment of Autism Spectrum Disorder. Neurotherapeutics.

[95] Asadabadi M., Mohammadi M.R., Ghanizadeh A., Modabbernia A., Ashrafi M., Hassanzadeh E., Forghani S., Akhondzadeh S. (2013). Celecoxib as adjunctive treatment to risperidone in children with autistic disorder: A randomized, double-blind, placebo-controlled trial. Psychopharmacology.

[96] Manter M.A., Birtwell K.B., Bath J., Friedman N.D.B., Keary C.J., Neumeyer A.M., Palumbo M.L., Thom R.P., Stonestreet E., Brooks H. (2025). Pharmacological treatment in autism: A proposal for guidelines on common co-occurring psychiatric symptoms. BMC Med..

[97] Bolandparvaz A., Harriman R., Alvarez K., Lilova K., Zang Z., Lam A., Edmiston E., Navrotsky A., Vapniarsky N., Van De Water J. (2019). Towards a nanoparticle-based prophylactic for maternal autoantibody-related autism. Nanomedicine.

[98] Neuchat E.E., Bocklud B.E., Kingsley K., Barham W.T., Luther P.M., Ahmadzadeh S., Shekoohi S., Cornett E.M., Kaye A.D. (2023). The Role of Alpha-2 Agonists for Attention Deficit Hyperactivity Disorder in Children: A Review. Neurol. Int..

[99] Loe I.M., Blum N.J., Shults J., Barbaresi W., Bax A., Cacia J., Deavenport-Saman A., Friedman S., LaRosa A., Mittal S. (2023). Adverse Effects of alpha-2 Adrenergic Agonists and Stimulants in Preschool-age Attention-deficit/Hyperactivity Disorder: A Developmental-Behavioral Pediatrics Research Network Study. J. Pediatr..

[100] Kaye A.D., Green A.M., Claude J.T., Daniel C.P., Cooley J.F., Sala K.R., Potharaju P., Rieger R., Patil S., Ahmadzadeh S. (2024). Efficacy and Safety of Alpha-2 Agonists in Autism Spectrum Disorder: A Systematic Review. Adv. Ther..

[101] Connor D.F., Findling R.L., Kollins S.H., Sallee F., Lopez F.A., Lyne A., Tremblay G. (2010). Effects of guanfacine extended release on oppositional symptoms in children aged 6–12 years with attention-deficit hyperactivity disorder and oppositional symptoms: A randomized, double-blind, placebo-controlled trial. CNS Drugs.

[102] Cinnamon Bidwell L., Dew R.E., Kollins S.H. (2010). Alpha-2 adrenergic receptors and attention-deficit/hyperactivity disorder. Curr. Psychiatry Rep..

[103] Dell’Osso L., Bonelli C., Giovannoni F., Poli F., Anastasio L., Cerofolini G., Nardi B., Cremone I.M., Pini S., Carpita B. (2025). Available Treatments for Autism Spectrum Disorder: From Old Strategies to New Options. Pharmaceuticals.

